# Enhancing Molecular High-Pressure Simulations by Implicit Solvation

**DOI:** 10.1021/acs.jpca.6c02133

**Published:** 2026-05-26

**Authors:** Nico Kißing, Felix Zeller, Tim Neudecker

**Affiliations:** † University of Bremen, 9168 Institute for Physical and Theoretical Chemistry, Leobener Straße 6, Bremen D-28359 Germany; ‡ Bremen Center for Computational Materials Science, University of Bremen, Am Fallturm 1, Bremen D-28359 Germany; § MAPEX Center for Materials and Processes, University of Bremen, Bibliothekstraße 1, Bremen D-28359 Germany

## Abstract

Gaussians On Surface Tesserae Simulate HYdrostatic Pressure (GOSTSHYP) and the eXtended Hydrostatic Compression Force Field (X-HCFF) are quantum chemical methods to simulate the effects of pressure on a single molecule. Both methods have the usage of discretized molecular surfaces in common, which are also needed in implicit solvation models like the Conductor-like Polarizable Continuum Model (C-PCM). However, a combined usage of GOSTSHYP or X-HCFF with C-PCM was not possible in previous implementations inside the Q-Chem program package. To address this circumstance, we present an independent surface construction routine for both of the pressure models. This routine enables a stable combination of C-PCM with GOSTSHYP or X-HCFF, which serves as the first step to consider the chemical surrounding inside these two pressure models. For three different compounds occurring in both neutral and zwitterionic forms, the energetic difference between these states under pressure via GOSTSHYP was investigated. Especially for compounds occurring in both zwitterionic and neutral structures, the C-PCM is essential to access the zwitterionic state. Calculated pressure dependencies in the Raman spectra of the zwitterionic structure of glycine show good agreement with experimental data. The dimerization reaction of orthosilicic acid at elevated pressure is also influenced by implicit solvation, leading to better agreement between the simulations and the experimental data. This study paves the way for the inclusion of explicit solvation to disentangle intra- and intermolecular effects that cause geometric and spectroscopic changes under pressure applied with GOSTSHYP and X-HCFF.

## Graphical abstract

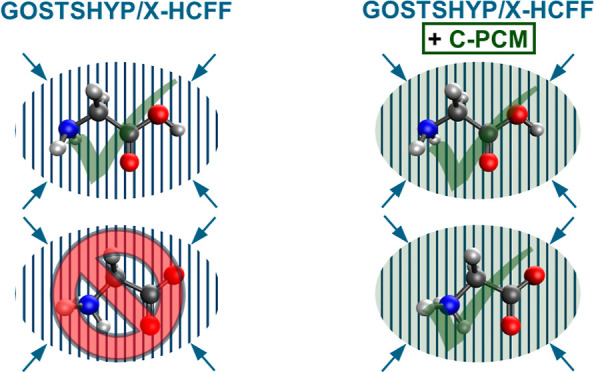

## 1 Introduction

Understanding the effects of extreme conditions on materials, for example, high-energy radiation^1^ or high temperature,^2,3^ is crucial to expand the knowledge about processes in which materials undergo substantial stressors. This is relevant in a variety of scientific disciplines, for example, in chemical engineering^4,5^ or in Earth science,^6,7^ as well as in astronomy.^8,9^ In this context, it is essential to also consider the influence of high pressure.^8,9^ Compounds can react to pressure with phase transitions, resulting in drastically changed properties of the material.^10–12^ A commonly known case is the pressure-induced transition of graphite to diamond.^13–15^ The occurrence of new phases is not limited to solid compounds and can also be observed in compounds that are gaseous under ambient conditions. For instance, a metallic phase of hydrogen is assumed to exist inside the planet Jupiter.^16^

The experimental investigation of materials under pressure is possible up to multiple hundreds of gigapascals using a diamond anvil cell, providing valuable information about structural and spectroscopic changes in samples.^17^ However, experiments can only contribute indirect insights into pressure-induced changes at a molecular level. Molecular responses to pressure can include conformational changes,^18^ chemical reactions,^19,20^ or spin-crossover in transition-metal complexes.^21,22^ As a different approach, theoretical methods are a useful tool to investigate these energetic and geometric changes in molecules due to pressure. Thus, they can complement experimental results and even make statements about the behavior of molecules in pressure regions that are experimentally not accessible yet.^23^

Pressure does not appear explicitly in any term of the electronic Hamiltonian.^23^ As a consequence, indirect quantum chemical methods for simulating pressure had to be designed. In the case of crystalline compounds for which periodic boundary conditions can be used, the volume of the unit cell can be reduced to mimic hydrostatic pressure.^21^ Molecular dynamics^24,25^ and especially ab initio molecular dynamics methods are also suitable for applying pressure on molecular systems and have been used frequently in a wide range of application fields.^26–30^

For the investigation of single molecules in this context, the eXtreme Pressure Polarizable Continuum Model (XP-PCM)^31–38^ was introduced as one of the first pressure models. The XP-PCM has its roots in the Polarizable Continuum Model (PCM), which enables the inclusion of implicit solvent effects in quantum chemical calculations.^39,40^ The molecule or atom is treated as a solute inside a solvent medium, which applies pressure using a Pauli repulsion term.^31,32^ This repulsion increases as the cavity volume decreases and becomes the predominant energetic factor.^32^

Mechanochemical models are an alternative way to simulate pressure by using forces that act on a molecule. Two of these methods are the Generalized Force-Modified Potential Energy Surface (G-FMPES) model^41^ and the Hydrostatic Compression Force Field (HCFF),^22^ adding forces to the nuclear gradient. The eXtended Hydrostatic Compression Force Field (X-HCFF)^42^ overcomes the limitation of both previously mentioned models that noncentrosymmetric molecules converge into spherical geometries as the forces act toward the centroid of the molecules.^22,41^ The X-HCFF allows the application of hydrostatic pressure by ensuring perpendicularly acting forces on the molecular surface.^42^ Additionally, pressure-induced changes in vibrational spectra can be investigated by calculating the analytic Hessian in X-HCFF.^43^

However, mechanochemical methods do not take the compression of the electron density of an atom or molecule directly into account.^42^ Another model affecting the electron density directly during the pressure application is the Gaussians On Surface Tesserae Simulate HYdrostatic Pressure (GOSTSHYP) approach.^20^ Gaussian potentials located on the molecular or atomic surface transfer the pressure to the system. Analogous to the XP-PCM, pressure-induced effects on the energy of molecular systems can be analyzed using GOSTSHYP.^20^ Unlike mechanochemical models that are strictly variations of geometry optimizations, GOSTSHYP can act as a geometry optimization method but is not limited to this usage. Both mechanochemical methods and GOSTSHYP frequently use density functional theory (DFT)^44,45^ as the underlying electronic structure method.

In previous implementations of X-HCFF and GOSTSHYP available in the quantum chemistry program Q-Chem,^46^ both methods could not be combined in a stable way with implicit solvation models like the Conductor-like Polarizable Continuum Model (C-PCM).^47,48^ This resulted from the simultaneous usage of algorithms handling the discretization of the molecular surface by one of the pressure models and C-PCM. Therefore, both pressure models were not capable of taking the chemical environment of a molecule into account in an implicit way. As a first step to include solvation effects in X-HCFF and GOSTSHYP, we present a new implementation for the handling of molecular surfaces in these models. This workflow is based on the reliable routine used in the PCM implementation of Q-Chem^49–51^ but enables an independent handling of molecular surfaces inside the pressure models. Moreover, the independent discretization routine allows the combination of X-HCFF and GOSTSHYP with implicit solvation models like the C-PCM to analyze the effects of pressure on solvated systems with these methods.

For instance, the investigation of zwitterionic structures with quantum chemical methods often proves to be a challenging task without the usage of additional models.^52^ In particular, the zwitterionic form of glycine is not accessible in gas phase calculations, which is reasonable due to its nonoccurrence in the gas phase.^52,53^ However, the zwitterionic form of glycine is dominant in aqueous solution at ambient conditions^53^ and in the solid state.^54–57^ Thus, the presented combination of GOSTSHYP and X-HCFF with C-PCM improves the physical accuracy of the pressure models by stabilizing experimentally observed high-pressure structures. Moreover, charge-separated molecules represent a particularly sensitive class of systems because their large dipole moments and polarity lead to a delicate balance between intramolecular electrostatics and environmental response.^58–60^ The present approach enables these effects to be explored systematically under hydrostatic pressure. This includes the investigation of pressure-induced effects on Raman shifts using GOSTSHYP, which are reported for the first time to the best of our knowledge.

In addition to simulations of zwitterionic systems, in this study, the effects of combining C-PCM with GOSTSHYP were also investigated for the dimerization of orthosilicic acid. To gain a deeper understanding of transport processes of liquids and solids in the crust and mantle of the Earth, the composition of silicates under pressure is one significant key.^61,62^ In the past, different experimental investigations using aqueous solutions of silicates have been performed.^61–64^ In addition to that, a variety of theoretical studies have been carried out.^65–69^ However, the intense research interest in this topic results in a diversity of findings, especially in the case of elevated pressure. Considering the complexity of the system, in which many factors like temperature or pH value influence its properties,^61^ a comparison of these studies is challenging. Here, we focus on the effects of pressure on the energetics of the dimerization of orthosilicic acid.

The paper is structured as follows: first, we give a short introduction to the theory behind X-HCFF (Section 2.1) and GOSTSHYP (Section 2.2) before the workflow of the new implementation is presented (Section 2.3). After the specification of the computational details (Section 3), the new routine is verified compared to the previous workflow to ensure the consistency of the new implementation (Section 4.1). Subsequently, we present three cases in which the usage of C-PCM has a significant impact on the geometries and energies of the molecules (Section 4.2). Also, we present the results for the dimerization reaction of orthosilicic acid, in which the inclusion of solvent effects changes the resulting structures (Section 4.3). A conclusion and an outlook on future research complete the paper (Section 5).

## 2 Theory

The section briefly summarizes the most important aspects of the theory behind the two pressure models X-HCFF and GOSTSHYP that were used in the performed calculations presented in this paper.

### 2.1 The eXtended Hydrostatic Compression Force Field (X-HCFF)

The mechanochemical X-HCFF allows the investigation of geometrical and vibrational changes of molecular systems due to pressure without the requirement of significantly higher computational resources than that for a gas phase calculation. In this approach, the molecular surface is computed as a van der Waals (vdW) surface and discretized using the Lebedev quadrature scheme.^70–72^ For each resulting tessera *j* located on the vdW sphere of an atom *i*, a force

1 $$f_{i,j}=-P\cdot a_{j}\cdot \frac{r_{i}-r_{j}}{\left|r_{i}-r_{j}\right|}$$

is defined as a product of the input pressure *P*, the area of the tessera *a*_*j*_, and the normal vector constructed by the positions of tessera **r**_*j*_ and the corresponding atom **r**_*i*_. The resulting force for one atom

2 $$f_{i}=\sum_{j}^{N_{Tess,i}}f_{i,j}$$

is the sum of all forces acting from the tesserae located on the vdW sphere of the atom. Uniform hydrostatic pressure is achieved by eliminating tesserae between adjacent atoms. However, this force definition does not exclude translations and rotations unambiguously. For that reason, a correction term

$$f_{corr}=-P\cdot \frac{1}{N_{Atoms}}\overset{N_{Tess}}{\underset{j}{\sum }}a_{j}\frac{r_{i}-r_{j}}{\left|r_{i}-r_{j}\right|}$$ 3

depending on the total number of atoms *N*_Atoms_, is introduced, which is subtracted from eq 2 for every atom. The calculated forces are then used to manipulate the nuclear gradient during geometry optimization. Besides the input pressure, users can also choose a factor that scales the used vdW radii to construct a Solvent-Accessible Surface (SAS).^73,74^ Additionally, the number of Lebedev tessellation points per atom is customizable. In a further development, the dependence on the scaling factor in X-HCFF was reduced by introducing a rescaling formalism.^75^ The electronic structure can be computed with wave function-based methods as well as DFT.

### 2.2 The Gaussians On Surface Tesserae Simulate HYdrostatic Pressure (GOSTSHYP) Approach

The GOSTSHYP method uses Gaussian potentials to affect the electron density as a result of pressure. The molecular surface is discretized, similarly to X-HCFF, as a Lebedev grid. On each tessera at position **r**_*j*_, a potential

4 $$G_{j}\left(r\right)=p_{j}\cdot \exp\left(-w_{j}{\left(r-r_{j}\right)}^{2}\right)$$

is defined containing an amplitude *p*_*j*_ and a width parameter *w*_*j*_. To ensure the reliable application of hydrostatic pressure, a smooth field of Gaussians needs to be generated. This is achieved by selecting a proper width parameter

5 $$w_{j}=\frac{\pi \;\ln2}{a_{j}}$$

for each Gaussian using the corresponding area of tessera *a*_*j*_. A detailed derivation of eq 5 can be found in the original publication.^20^

Convergence between the compressed electron density and the acting pressure is achieved if the corresponding forces cancel out each other. The inner force results from the compression of the electron density, while the outer force is caused by pressure. Using the definition of pressure as the normal force per area, an outer force for each tessera can be defined as

$$F_{outer,j}=P\cdot a_{j}$$ 6

based on the input pressure *P* and the area of the tessera. To calculate the inner force, the energetic contribution *E*_p_ from the pressure application needs to be evaluated. By considering the molecular orbitals {ϕ_*p*_} and the electronic one-particle density matrix *D*_*pq*_, the energy contribution needs to be computed according to

$$E_{p}=\underset{j}{\sum }\underset{pq}{\sum }D_{pq}\left\langle \phi_{p}\left|G_{j}\left(r\right)\right|\phi_{q}\right\rangle$$ 7

via one-electron three-center overlap integrals in which the Gaussian is an s-type function. The inner force acting perpendicularly to the surface of the molecule is the scalar product of the derivative vector of the energy contribution and the surface normal vector **n**, yielding

$$F_{inner,j}=\frac{\partial E_{p}}{\partial x_{j}}n_{x}+\frac{\partial E_{p}}{\partial y_{j}}n_{y}+\frac{\partial E_{p}}{\partial z_{j}}n_{z}\;.$$ 8

For more detailed information about the computation of the required mathematical expressions used in GOSTSHYP, the interested reader is kindly referred to the initial publication.^20^ Similar to X-HCFF, a scaling factor, here named as the Tessellation Sphere Scaling Factor (TSSF), and the number of tessellation points can be chosen by the user. By implementing an integral-screening algorithm in Q-Chem, the evaluation of the one-electron three-center overlap integrals has become less computationally demanding, with a scaling behavior of $O\left(N_{Tess}\right)$ .^76^ Recently, GOSTSHYP was implemented in the Turbomole^77–79^ program package using an integral-direct algorithm.^80^ The electronic structure can be calculated on a Hartree–Fock (HF)^81–83^ or DFT level of theory as GOSTSHYP interacts with the Self-Consistent Field (SCF) algorithm of the quantum chemical program. During one SCF cycle, the electron density and the amplitudes *p*_*j*_ are optimized and self-consistent in the end.

### 2.3 The New Surface Construction and Tessellation Routine

The stable combination of X-HCFF and GOSTSHYP with PCM in Q-Chem has required the implementation of an independent routine for constructing and tessellating the molecular surface of the desired system. In all previous implementations of both methods in Q-Chem, X-HCFF and GOSTSHYP accessed the routine used for PCM. If PCM or one of the pressure models is used individually, then the existing routine has proven to be reliable. However, a combined usage of one pressure model with PCM can lead to instabilities of the program or uncertain results, which is caused by unintentionally overwritten temporary files.

To overcome this circumstance, the pre-existing PCM surface class was used as a basis for building an independent surface class, which is accessed exclusively by one of the pressure models. In that way, a solid basis was established, which ensures that no undesired interaction with other functionalities of Q-Chem occur. The general workflow of the new way of how information about the molecular surface is saved is shown in Figure 1. After the initialization of either the X-HCFF or GOSTSHYP class (1), the Tess class is instantiated (2). It is a base class allowing different ways of constructing molecular surfaces. Via the new keyword “tess_method” in the $distort section of an input file, the user can choose which routine is accessed for the tessellation of the surface (3). At the moment, three options are available: the previous way, which was implemented as a part of PCM, is initialized if the “tess_method” keyword is set to “scrf”, as it was implemented in the libscrf library of Q-Chem. In contrast, the new independent routine is accessed if the option “distort” is selected as the new routine, as it is located in the libdistort library in Q-Chem. In this library, all source codes regarding the pressure models are stored. The “occ” option refers to the usage of a vdW surface with an Outer Cavity Correction (OCC), which has already been available in Turbomole and improves the SCF convergence.^80^

**Figure 1:**
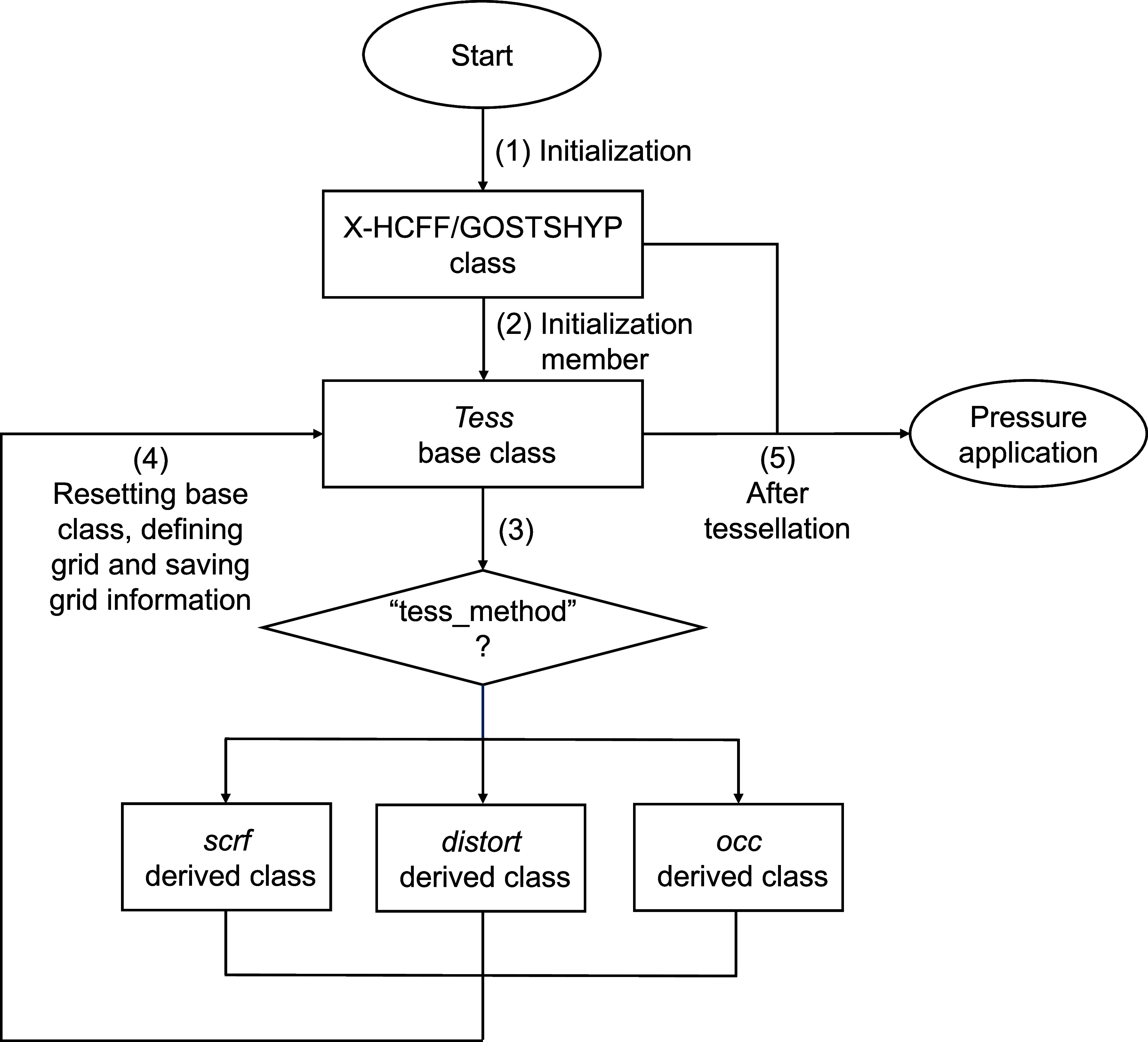
Workflow representing the new handling of molecular surfaces inside the X-HCFF and GOSTSHYP approaches in Q-Chem.

After checking which of the available discretization routines should be used, the base class is reset, and the surface grid is constructed and then saved into the base class (4). From the tessellation, the resulting surface normal vectors, areas, and area gradients are used for the pressure application. The “distort” and “scrf” ways of tessellation will return identical results if the default vdW surface or SAS is used. Other cavity options, like the Solvent-Excluded Surface (SES),^84,85^ are only available in the pre-existing “scrf” option.

The new option to choose different discretization algorithms is also the basis for introducing new ways of handling the molecular surfaces. This will allow more flexibility and control over how molecular surfaces are computed inside the pressure models. The first application is the aforementioned vdW surface with an OCC. The new routine for the selection of different cavity construction algorithms is publicly available as part of Q-Chem 6.3.1.

## 3 Computational Details

The independent tessellation routine for X-HCFF and GOSTSHYP was implemented in a locally changed development version of Q-Chem 6.2, which was used in all calculations in which C-PCM is combined with one of these pressure models. If not noted otherwise, default values were used in the quantum chemical calculations.

For verification purposes, energy and force calculations for X-HCFF, GOSTSHYP, or C-PCM were performed and compared with results using the release version of Q-Chem 6.0. The code verification of the independent surface tessellation routine was performed with calculations at the HF level of theory with the 6–31G^86^ basis set. In this context, the pressure models were used with two different pressure values (0 and 50 GPa) to ensure that only intended pressure application takes place. For C-PCM, two dielectric constants (1 and 1.001) were used, respectively. All possible combinations of pressure models and C-PCM with these values were performed.

In all combined applications of the pressure models with C-PCM, the “distort” option was chosen to ensure robust execution. To investigate the effects of pressure application via X-HCFF and GOSTSHYP on solvated molecules with C-PCM, the DFT level of theory with the B3LYP^87–90^ exchange–correlation functional, the D3­(BJ)^91,92^ dispersion correction, and the aug-cc-pVDZ^93,94^ basis set was chosen. All structures were optimized first without the influence of pressure. By performing vibrational frequency calculations, it was ensured that the optimized structures are minima on the potential energy surface. These were then used for the pressure application. For the investigation of zwitterionic structures, GOSTSHYP was used due to its ability to calculate the energetic contribution of pressure. The choice of the dielectric constants is explained later on. Molecular volumes were obtained as vdW volumes by performing single-point calculations using the PV (pressure times volumes) method^95^ with 1202 tessellation points per atom. Raman spectra of zwitterionic glycine under pressure were calculated on the same level of theory with GOSTSHYP or X-HCFF combined with C-PCM. These were compared with experimental data, which were extracted by using the WebPlotDigitizer software.^96^ For the dimerization of orthosilicic acid, the 6–311++G­(d,p)^97,98^ basis set was used with a TSSF of 1.8 in GOSTSHYP calculations. The critical points of the potential energy surface were verified by vibrational frequency analysis. Due to deactivated symmetry detection in PCM calculations, the symmetry was ignored in all pressure calculations.

## 4 Results and Discussion

First, the introduced surface construction routine for X-HCFF and GOSTSHYP is tested against the existing algorithm used in PCM (Section 4.1). Subsequently, the combined usage of X-HCFF and GOSTSHYP with C-PCM is tested for glycine, sulfamic acid, and 2-dimethylaminobenzoic acid, which can occur in both a neutral state and a zwitterionic state (Section 4.2). The latter is characterized by the presence of positively and negatively charged functional groups within an overall neutral molecule. After that, the results for the dimerization reaction of orthosilicic acid are presented (Section 4.3).

### 4.1 Code Verification

The consistency of the independently working surface construction routine was verified with comparative energy and force calculations of water, ammonia, and benzene in different combinations of X-HCFF or GOSTSHYP with C-PCM. On the one hand, the new implementation should match the results of the established one to machine-level accuracy. On the other hand, the new routine has to enable a stable usage of C-PCM combined with one of the pressure models.

First, it was ensured that the pressure models do not manipulate results unintentionally. For that reason, X-HCFF and GOSTSHYP with the selected “distort” option were tested with a pressure value of 0 GPa. The quantities of energy and the Root Mean Square (RMS) gradient, which is the square root of the average squared Cartesian force components acting on the atoms of a molecule, did not change compared to a regular calculation without including the models. The detailed listing of all values can be found in the Supporting Information, in which Tables S1 to S3 show the energy values for water, ammonia, and benzene, while Tables S4 to S6 show the corresponding RMS gradients. After that, the consistency of the results for the isolated usage of PCM, GOSTSHYP, and X-HCFF with both “scrf” and “distort” options as well as Q-Chem 6.0 could be verified for different pressure values (0 and 50 GPa) or dielectric constants (1 and 1.001), respectively.

With additional usage of C-PCM (ϵ = 1) for X-HCFF (0 GPa), the energy and gradient values also show no difference, regardless of which tessellation routine or Q-Chem version was chosen. However, if GOSTSHYP was requested, the calculations crashed when using the “scrf” routine, which is caused by the manipulation of the SCF algorithm of both PCM and GOSTSHYP. In that case, due to the usage of the same code for the surface construction, the two models interfere in an undesirable way. Moreover, a dielectric constant higher than 1 results in a further malfunction of Q-Chem using default values without the new implementation. On that condition, the PCM gradient needs to be calculated, in which PCM and the pressure models overwrite temporary files causing instabilities of the program.

Only the newly introduced surface construction routine provided a stable way to combine one of the pressure models with C-PCM, regardless of the chosen pressure values or dielectric constants. The findings of the manually performed code verification were also supported by executing specific unit tests in which the new implementation is tested compared with reference data. Again, all testing confirmed the successful implementation of an independent surface construction routine for GOSTSHYP and X-HCFF.

### 4.2 Zwitterionic Structures under Pressure

#### 4.2.1 Glycine

To enable the investigation of pressure on the zwitterionic structure of glycine with GOSTSHYP on a single-molecule level, C-PCM was included in the quantum chemical calculations. The requirement of using C-PCM to receive the zwitterionic form of glycine^52,53^ or small proteins^99^ as a stable structure is well-known in the literature. This behavior was consistently observed in our calculations for glycine, as well. Moreover, it was demonstrated that C-PCM improves the SCF convergence of charge-separated molecules significantly.^100^

In this work, the energetic difference between the zwitterionic and neutral state depending on pressure is of special interest. On the reoptimized initial structures obtained from Boča et al.,^57^ pressure was applied up to 300 GPa via GOSTSHYP including C-PCM. This pressure region is experimentally difficult to reach.^17^ However, multiple theoretical studies were performed in the past in which the behavior of different materials under such high pressure was simulated.^101–103^ Especially in the context of astronomy, the investigation of these high pressures provides valuable insight into planetary processes and compositions.^104^ Therefore, the application of GOSTSHYP, even in these extreme conditions, can lead to additional knowledge about the general properties of materials. The corresponding structures of the neutral and zwitterionic state are shown in Figure 2, combined with all other structures examined later on.

**Figure 2:**
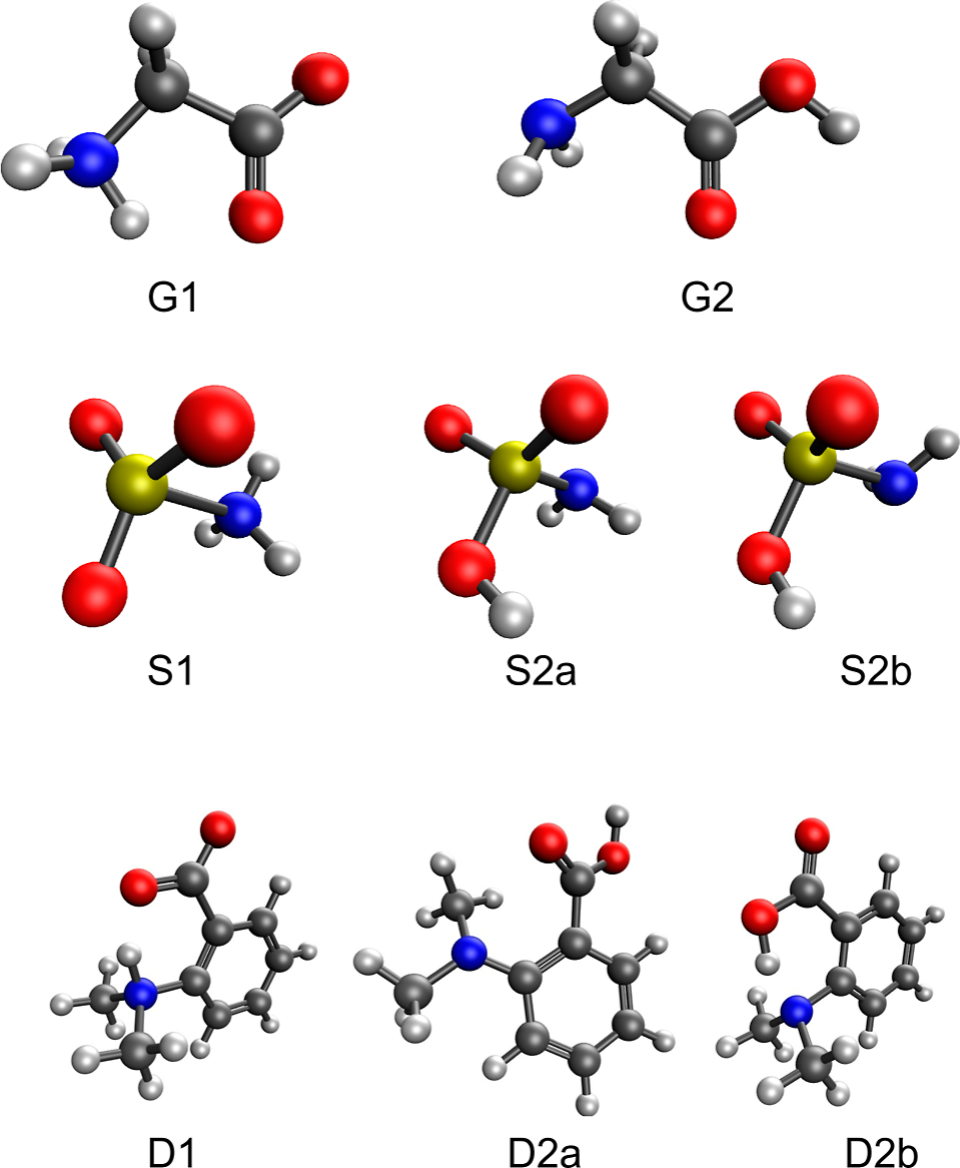
Structures of the zwitterionic state (left) and of the neutral state (right) of glycine (G1 and G2), sulfamic acid (S1, S2a, and S2b), and 2-dimethylaminobenzoic acid (D1, D2a, and D2b) optimized on a DFT level of theory (B3LYP-D3­(BJ)/aug-cc-pVDZ). C-PCM was included in the calculations with the dielectric constant of water available in Q-Chem. Carbon atoms are shown in dark gray, nitrogen atoms are shown in blue, oxygen atoms are shown in red, sulfur atoms are shown in yellow, and hydrogen atoms are shown in light gray.

When including C-PCM in pressure simulations, a nontrivial question is the choice of the dielectric constant. Under pressure, not only do the properties of the molecule of interest change, but so do those of the surrounding environment. The dielectric constant of water is not well-understood in the high-pressure regime.^105,106^ Experimental results are limited to pressure values of 500 MPa.^107^ However, both experimental^107^ and theoretical studies^105,106^ suggest that the dielectric constant increases with increasing pressure and constant temperature. To test the influence of an increasing dielectric constant, the calculations for glycine were performed with a logarithmic extrapolation of the pressure-dependent dielectric constant of water at 298 K by Floriano and Nascimento.^107^ The results were then compared to calculations in which the dielectric constant of water under ambient conditions was used.

For pressures up to 200 GPa, clear discrimination between the two different states of glycine is possible. For these pressure values, the energetic differences between the states are shown in Table 1.

**Table 1:** Energetic Differences Δ*E* between the Neutral and Zwitterionic State of Glycine under Hydrostatic Pressure Applied with the GOSTSHYP Method on a DFT Level of Theory (B3LYP-D3­(BJ)/aug-cc-pVDZ)^a^

	without pressure	100 MPa	1 GPa	10 GPa	100 GPa	200 GPa
						
Δ*E* / (kcal/mol)	4.284	4.315	4.516	5.569	13.73	19.67
Δ*E*(ϵ) / (kcal/mol)	–	4.325	4.593	5.722	13.91	19.82
Δ(Δ*E*) / (kcal/mol)	–	0.010	0.077	0.153	0.17	0.16

a For the stabilization of the zwitterionic structure, C-PCM was used. Positive values indicate the energetic preference of the zwitterionic structure. The differences Δ­(Δ*E*) between the usage of the pressure-independent (Δ*E*) and -dependent (Δ*E*(*ϵ*)) dielectric constant of water extrapolated by Floriano and Nascimento are also presented.

Comparing the results of the two different approaches for setting the dielectric constant, it can be noted that using the extrapolated pressure-dependent dielectric constant affects the energies only weakly. Due to the uncertainty in how the dielectric constant of water changes upon the application of pressure higher than 500 MPa, which is given in the discussed calculations, it was decided to use the pressure-independent dielectric constant in all further calculations. At this point, we emphasize the explorative character of the performed calculations. It is expected that the continuum approximation loses accuracy in the high-pressure regime due to structural changes of water.^108–112^ However, as the stabilization of zwitterionic structures under these high-pressure conditions is the main focus of this work, using C-PCM provides a reliable way of accessing those structures in an inexpensive way.

Another important aspect to address is the possible usage of two independent cavities for the pressure model and C-PCM, respectively. With the compression of the geometry due to pressure, the size of the cavity decreases as the cavity is reconstructed at every step of the geometry optimization. This resulted in a slightly reduced cavity size. C-PCM only contributes to the electrostatic polarization due to the dielectric medium, with no additional compression term included.^47,48^ With the exception of a pressure-dependent dielectric constant, it is not expected that the use of two cavities leads to significant mutual interactions. In contrast, a further shrinkage of the cavity by decreasing its scaling factor with pressure could introduce an additional energetic contribution that may become disproportionately large. In that case, an already compressed structure due to GOSTSHYP could increase unphysically in energy because of the penetration caused by the dielectric. Thus, the chosen approach expands the number of systems that can be investigated under high pressure with the aforementioned pressure models.

For glycine, the zwitterionic state is more stable in the absence of pressure and at all applied pressure values. Furthermore, the stabilization increases with increasing pressure, which can be explained with the compactness of the zwitterionic structure. The molecular volumes of the zwitterionic and neutral structures of glycine are shown in Figure 3. At all pressure values with a distinguishable structure of glycine, the zwitterionic structure occupies less space than the neutral structure, which is favored at elevated pressure.

**Figure 3:**
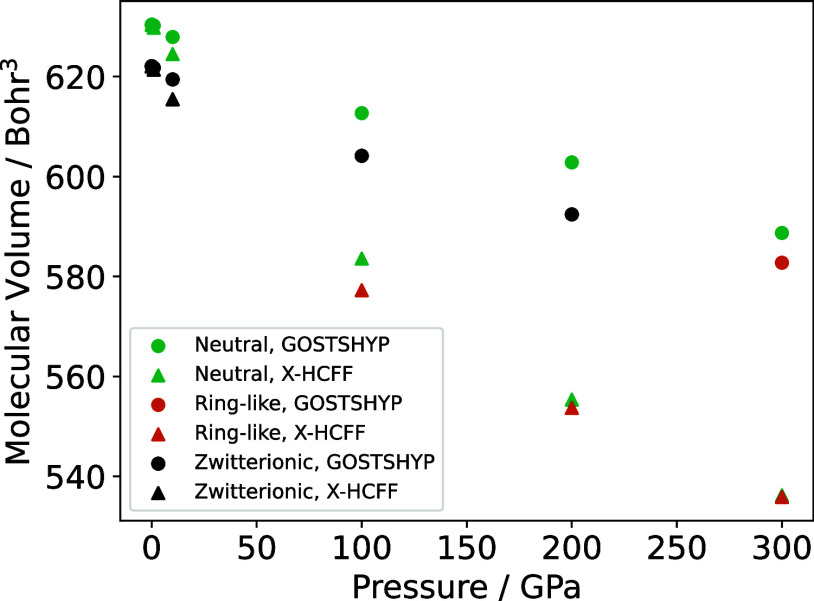
Molecular volumes of the three different structures of glycine (neutral, ring-like, and zwitterionic) depending on the applied pressure via GOSTSHYP and X-HCFF, respectively, using the pressure-independent dielectric constant of water.

In addition to the presented results obtained with GOSTSHYP, X-HCFF was also used to compress glycine under hydrostatic pressure. As the energy values of the compressed structures are only influenced indirectly by the distorted geometry in X-HCFF, the comparison of GOSTSHYP and X-HCFF is focused on the molecular volumes of glycine under pressure. At the same pressure values, the use of X-HCFF leads to more compact and distorted structures than GOSTSHYP. Moreover, the differences in volume for the neutral and ring-like structures become less pronounced with increased pressure. As this work focuses more on the energetic influences of pressure simulations coupled with C-PCM, GOSTSHYP is used exclusively in all other cases.

At higher pressures, the zwitterionic structure converges into a ring-like structure, which can be understood as a transition from the zwitterionic state toward a neutral conformation. Vibrational frequency analyses revealed that this ring-like structure is a minimum on the potential energy surface at 300 GPa using GOSTSHYP. In the case of X-HCFF, the ring-like structure is already detectable at 100 GPa. In contrast, a very similar but uncompressed structure represents the transition state of the intramolecular hydrogen transfer between the amino group and the carboxyl group under ambient conditions. This structure, as well as the structure obtained at 300 GPa with GOSTSHYP, is shown in Figure 4. The conversion of a transition state into a minimum on the potential energy surface by applying pressure was previously observed in a publication from Kumar et al.,^113^ in which X-HCFF was used to investigate the effects of hydrostatic pressure on a [2,3]-sigmatropic rearrangement.

**Figure 4:**
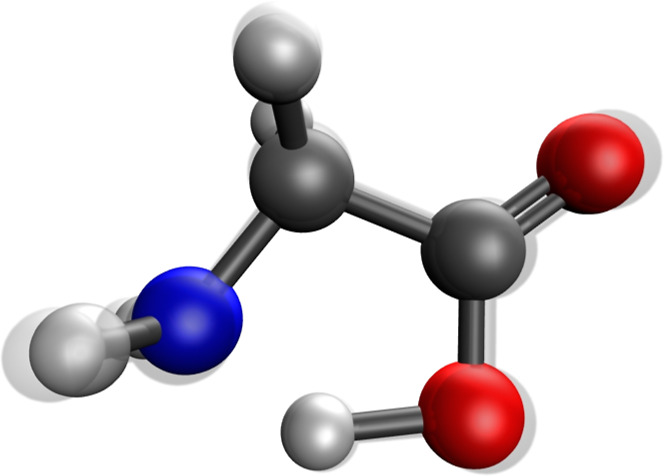
Ring-like structure of glycine obtained at 300 GPa using GOSTSHYP combined with C-PCM. The structure of the transition state at ambient conditions is represented as a shadowed structure within the overlay.

To compare the chosen approach with experimental data, Raman spectra of the zwitterionic form of glycine under the influence of pressure were calculated and compared to experimental data provided by Hinton et al.^56^ Besides the absolute values of the Raman shifts, their pressure dependence is of special interest. We focus on four modes occurring in the experimental and theoretical spectra with relatively high intensities, which are the C–C stretching mode, the CH_2_ scissors mode, the symmetric CH_2_ stretching mode, and the asymmetric CH_2_ stretching mode. All quantities of the pressure dependencies of the investigated Raman shifts can be found in Table 2. Additionally, the complete Raman spectra using GOSTSHYP and X-HCFF are visualized in the Supporting Information (Figures S1 and S2).

**Table 2:** Pressure Dependence dν/d*p* of the Raman Shift for the Four Vibrational Modes Calculated with GOSTSHYP or X-HCFF Combined with C-PCM on the B3LYP-D3­(BJ)/aug-cc-pVDZ Level of Theory Compared to Experimental Data, Extracted with Permission from Hinton et al.,^56^ Copyright 2019 Royal Society of Chemistry^a^

	Pressure dependence dν/d*p* / (cm^–1^ GPa^–1^)		
Vibrational mode	GOSTSHYP	X-HCFF	Experimental
C–C stretching	1.5	2.7	2.1
CH_2_ scissors	3.1	2.5	2.3
CH_2_ symmetric stretching (*p* < 27 GPa)	4.1	7.5	6.0
CH_2_ symmetric stretching (*p* > 27 GPa)	4.1	7.5	4.5
CH_2_ asymmetric stretching	4.2	7.4	7.2

a For both experimental and theoretical values, linear regression was used to determine the dependence up to 42 GPa. For the CH_2_ symmetric stretching vibration, two different pressure dependencies were identified for the experimental data with a pressure threshold of 27 GPa.

For the C–C stretching and CH_2_ scissors vibrational modes, the corresponding Raman shifts depending on pressure up to 42 GPa applied with GOSTSHYP or X-HCFF are shown in Figure 5.

**Figure 5:**
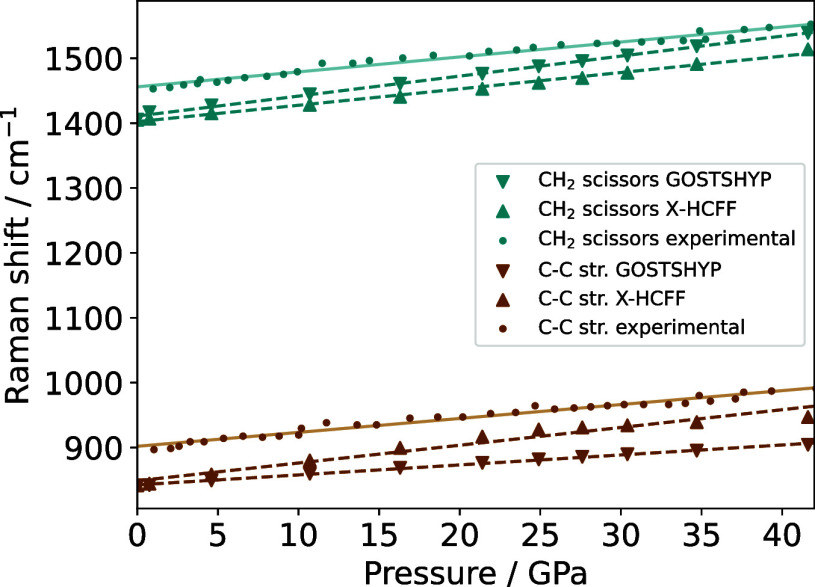
Raman shifts corresponding to the C–C stretching vibration (C–C str., brown) and CH_2_ scissors vibration (turquoise) depending on pressure applied by GOSTSHYP (inverted triangles) or X-HCFF (upright triangles) compared to experimental data, obtained with permission from Hinton et al.^56^ (dots), Copyright 2019 Royal Society of Chemistry. For all data, regression curves are shown.

For both vibrations, it can be noted that the linear increase of the Raman shifts with pressure (blue shift) can be reproduced. However, in the absence of pressure, the Raman shift is systematically underestimated with regard to experimental values.

In the case of the C–C stretching vibration, the pressure-induced blue shift by applying GOSTSHYP (1.5 cm^–1^ GPa^–1^) or X-HCFF (2.7 cm^–1^ GPa^–1^), respectively, is in reasonable agreement with the experimental value (2.1 cm^–1^ GPa^–1^). Regarding the Raman shift associated with the CH_2_ scissors vibration, the agreement of the absolute values increases for the GOSTSHYP model with pressure in the investigated pressure range. While the blue shift is overestimated in the case of GOSTSHYP (3.1 cm^–1^ GPa^–1^) compared to the experimental value (2.3 cm^–1^ GPa^–1^), X-HCFF shows a quantitative agreement with the experiment (2.5 cm^–1^ GPa^–1^). However, it has to be noted that deviations from experimental values on this scale can also be observed in the periodic pressure calculations performed by Hinton et al. This indicates that the discrepancy may be caused by the approximate nature of DFT.

In Figure 6, the pressure dependence of the Raman shift associated with the CH_2_ symmetric and asymmetric stretching vibration is shown. The aforementioned overestimation of the Raman shift at ambient conditions by DFT can be observed for the two modes as well but are comparable with the results obtained from the periodic calculations from Hinton et al. Both GOSTSHYP and X-HCFF show a linear pressure dependence for both modes and thus reproduce the experimentally observed blue shifts. However, the symmetric CH_2_ stretching mode shows a lower dependence at higher pressure. Under the assumption of linear regression, the experimental data can be handled best by performing two regressions with a pressure threshold of 27 GPa for this mode. While the results obtained with X-HCFF show a weaker similarity to the experimental data with increasing pressure, a contrary trend can be determined from the GOSTSHYP results. The absolute values become more similar with increasing pressure. Above 27 GPa, both the absolute values of the Raman shift and their pressure dependence are in quantitative agreement with the experimental data (4.1 cm^–1^ GPa^–1^ for GOSTSHYP and 4.5 cm^–1^ GPa^–1^ for the experiment).

**Figure 6:**
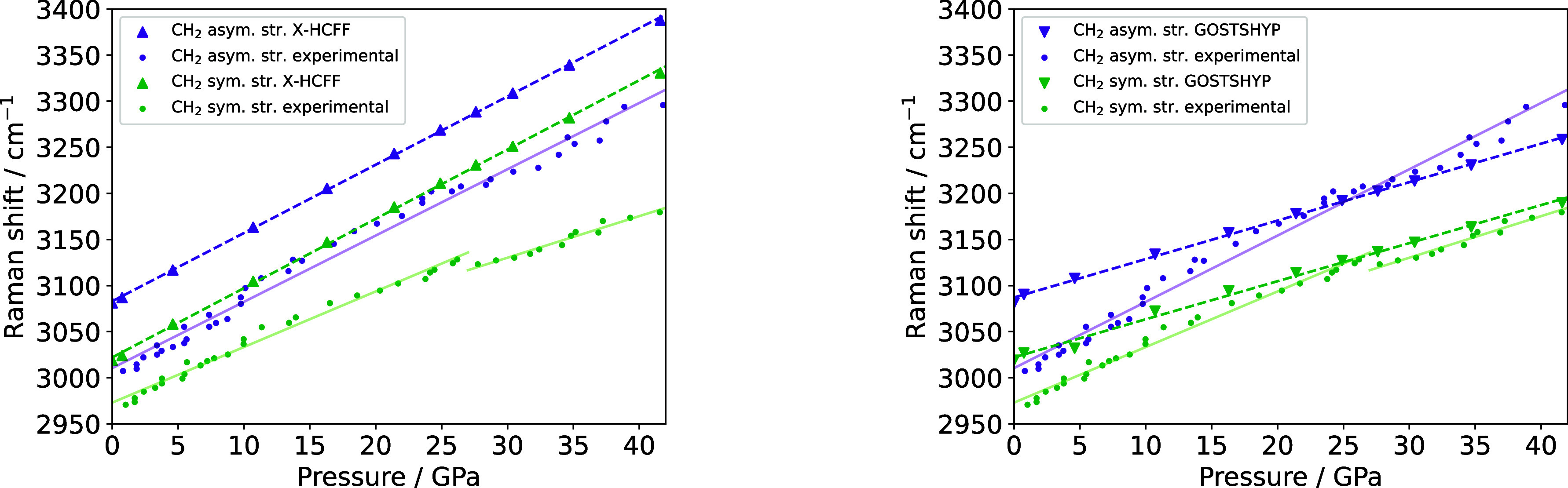
Raman shifts corresponding to the symmetric CH_2_ stretching (CH_2_ sym. str., green) and asymmetric CH_2_ stretching (CH_2_ asym. str., purple) vibration depending on pressure applied by X-HCFF (upright triangles, left) or GOSTSHYP (inverted triangles, right) compared to experimental data, obtained with permission from Hinton et al.^56^ (dots), Copyright 2019 Royal Society of Chemistry. For all data, regression curves are shown.

The Raman shift of the asymmetric CH_2_ stretching mode shows, like the other modes investigated in this work except for the symmetric CH_2_ stretching mode, a linear dependence on pressure (7.2 cm^–1^ GPa^–1^). Both models show similar values for the pressure dependence compared to the ones obtained for the symmetric one (4.2 cm^–1^ GPa^–1^ for GOSTSHYP and 7.4 cm^–1^ GPa^–1^ for X-HCFF). This indicates that the different Raman shifts for both modes are caused by intermolecular interactions as they are observable in the experimental data and the periodic DFT calculations. While the absolute values of the Raman shift with pressure applied by GOSTSHYP are in good agreement with the experiment at pressure values higher than 15 GPa, the pressure dependence is underestimated. In contrast, the pressure dependence is in quantitative agreement if X-HCFF is used. Overall, the presented results indicate that the single-molecule approach for the zwitterionic form of glycine can be used as a sophisticated model for its behavior under pressure. For this reason, the results suggest that the chosen approach is generally suitable for application to the other zwitterionic systems considered in this study. However, for vibrations in which intermolecular interactions play a significant role, like hydrogen bonds in the case of the N–H stretching modes, the single-molecule approach reveals some limitations.

#### 4.2.2 Sulfamic Acid

Another interesting example of compounds occurring in both neutral and zwitterionic states is sulfamic acid. It is an industrially relevant chemical compound used as a cleaning agent or recently in catalytic applications.^114,115^ Again, the zwitterionic form is preferred in the solid phase.^114^ For gas-phase calculations, it could be shown that the energetic differences are strongly dependent on the level of theory,^114^ indicating that the two possible states are energetically very similar. In this work, the same level of theory as that used for glycine was chosen. The geometries were obtained from the literature.^114,116^ For the neutral state, two conformers were considered. For each of the neutral conformers, the energetic difference between the neutral and zwitterionic states under pressure applied with the GOSTSHYP method was evaluated up to a pressure of 1000 GPa to assess pressure-induced trends. The energetic differences between the two neutral conformers and the zwitterionic state of sulfamic acid with an increase in pressure are shown in Figure 7.

**Figure 7:**
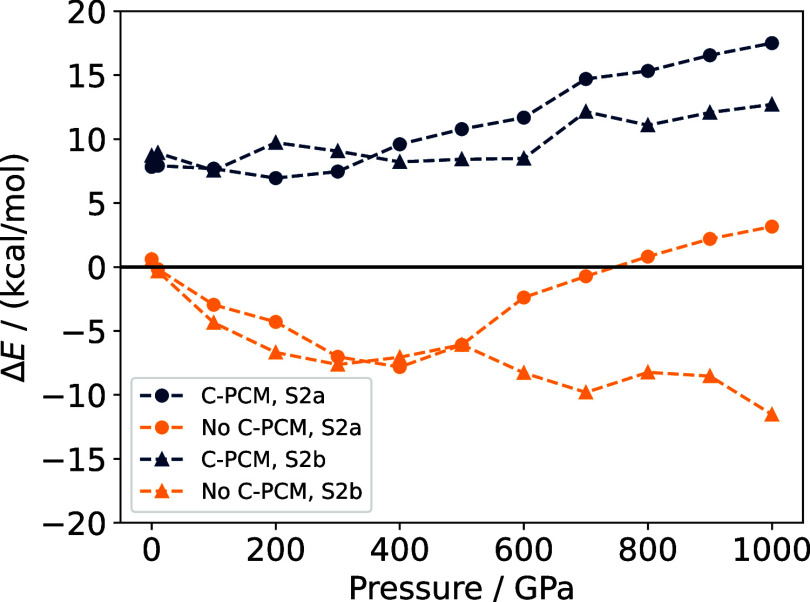
Pressure-dependent energetic difference between the neutral conformers S2a (circles) and S2b (triangles) and the zwitterionic state of sulfamic acid calculated with and without the usage of C-PCM on a DFT level of theory (B3LYP-D3­(BJ)/aug-cc-pVDZ). Pressure was applied using the GOSTSHYP method. Energetic differences for which C-PCM was used are visualized in blue, while results in the absence of solvation models are shown in yellow. At positive energy values, the zwitterionic state is energetically preferred.

It can be noted that the zwitterionic form of sulfamic acid is stable in the absence of an implicit solvation model. This allows the exact investigation of the effects of solvation in these pressure simulations. For both conformers and at all applied pressures, the use of C-PCM leads to a stabilization of the zwitterionic state over the neutral state. The amount of stabilization due to C-PCM is also pressure-dependent. For S2a, a small local minimum of stabilization can be detected at 200 GPa. At higher pressures, the stabilization increases almost linearly. For the neutral conformer S2b, a rather small pressure dependence can be detected.

In contrast, the energetic difference changes significantly with pressure if the calculations are performed in the absence of the C-PCM. Without pressure application via GOSTSHYP, the zwitterionic structure is slightly more stable. However, it was reported in previous computational studies that the energetic preference of the two different forms of sulfamic acid in the gas phase highly depends on the chosen level of theory.^114^ A detailed benchmark analysis using different levels of theory is not addressed within the confines of this study as we focus on the effects of pressure on molecules combined with implicit solvation.

For S2b, the neutral state becomes, in general, more stable under pressure if GOSTSHYP is used exclusively. On the other hand, the energetic difference between the neutral conformer S2a and the zwitterionic conformer depends significantly on the applied pressure. At 400 GPa, the stabilization of the neutral structure S2a reaches its maximum. In the pressure region between 300 and 400 GPa, the neutral state features a torsionally displaced amino group along the molecular axis. This is not detectable for the other conformer, nor with the use of C-PCM. This can be interpreted as an indicator that the inclusion of C-PCM in GOSTSHYP calculations contributes to the stabilization of the conformation during the geometry optimization, even if the structure is not zwitterionic. For higher pressures, the S2a isomer becomes less energetically preferred, and at pressures higher than 700 GPa, the zwitterionic structure is again more stable.

The results for sulfamic acid not only show that the C-PCM stabilizes the zwitterionic state compared to gas-phase calculations in a wide range of pressures but also show that implicit solvation has a significant impact on the overall trend in which the energetic difference changes due to pressure.

#### 4.2.3 2-Dimethylaminobenzoic Acid

The last example of compounds that can occur in both zwitterionic and neutral states presented in this work is 2-dimethylaminobenzoic acid.^117–120^ A potential pharmacological application of 2-dimethylaminobenzoic acid in the treatment of breast cancer has recently been proposed.^121^ Again, one zwitterionic and two low-energy conformers for the neutral state have been investigated as another test system to evaluate the effects of pressure on zwitterionic structures.

Similar to glycine, the zwitterionic state is not accessible with gas-phase calculations. Thus, C-PCM plays once again a crucial role in stabilizing the zwitterionic form observed in the solid state.^120^ For the neutral state, similar to that of sulfamic acid, two low-energy conformers were investigated. To stabilize the pressure application, the number of tessellation points was set at 302 in this case. The results for the energy differences of neutral and zwitterionic states under pressure are shown in Figure 8.

**Figure 8:**
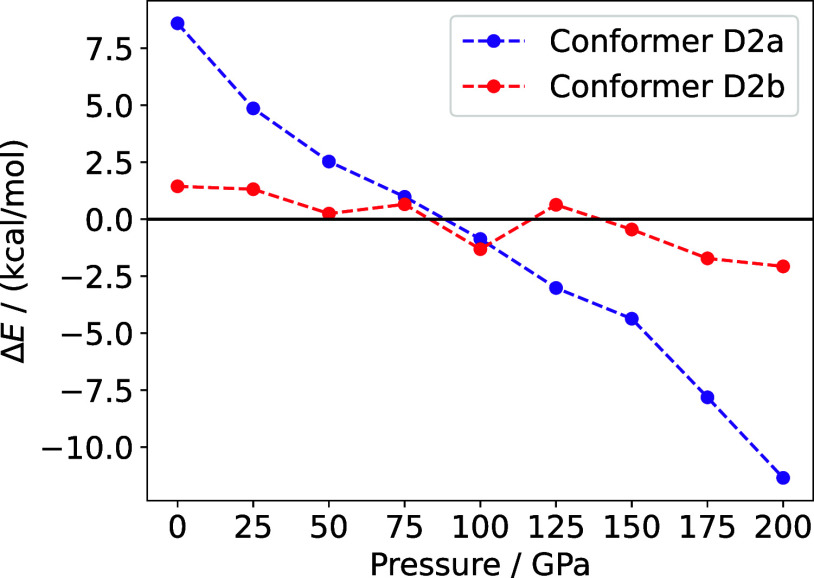
Energy differences between the zwitterionic and neutral state of 2-dimethylaminobenzoic acid under the influence of pressure applied via GOSTSHYP on a DFT level of theory (B3LYP-D3­(BJ)/aug-cc-pVDZ/C-PCM). For the neutral state, conformers D2a (violet points) and D2b (orange points) were investigated. At pressure values at which Δ*E* is positive, the zwitterionic state is preferred.

In the moderate-pressure region up to 75 GPa, the zwitterionic structure is preferred over both neutral conformers. However, the energetic difference between the states is significantly lower for the neutral conformer D2b than for conformer D2a up to 50 GPa. This can be explained by the greater structural similarity of D2b with the zwitterionic state.

For both neutral conformers, the zwitterionic state becomes less favored with increasing pressure. In the case of conformer D2a, the energetic difference decreases linearly with increasing pressure. For conformer D2b, the obtained Δ*E* values suggest a weak pressure dependence, and no consistent trend can be observed. Only at high pressure, a small decrease of Δ*E* can be identified with certainty. Compared with D2a, conformer D2b is less flexible under pressure due to the hydrogen bond between the carboxyl group and the dimethylamino group. Under pressure, D2a can change into a more planar conformation, which is validated by comparing the pressure dependence of the molecular volumes for the three different structures. These are shown in Figure 9. Conformer D2a shows the lowest molecular volume at all investigated pressure values. Especially in the high-pressure region, the higher compressibility of D2a is indicated by the stronger decrease of the volumes with the pressure compared to the two other structures.

**Figure 9:**
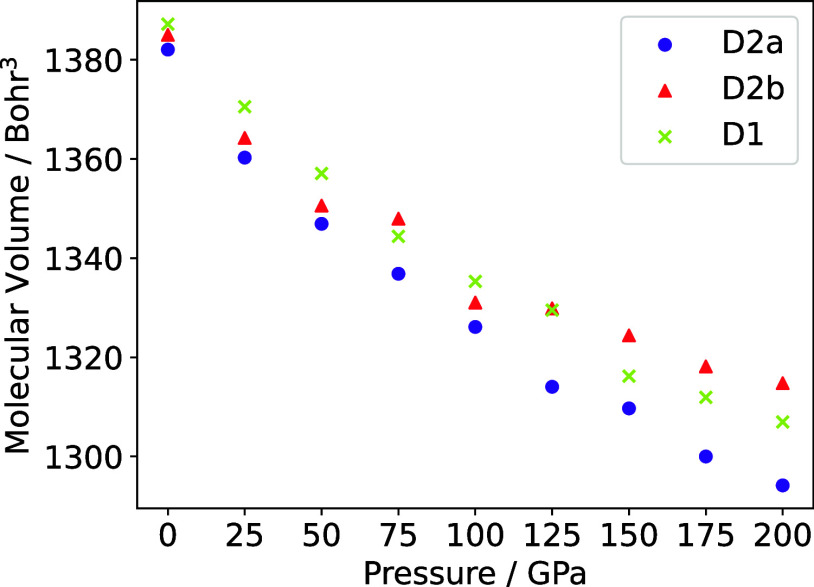
Molecular volumes of the three different structures of 2-dimethylaminobenzoic acid (D1, D2a, and D2b) depending on the applied pressure via GOSTSHYP using the pressure-independent dielectric constant of water.

As the Δ*E* values decrease linearly in the case of the neutral conformer D2a, the energetic preference changes between 75 and 100 GPa from the zwitterionic state to the neutral state. This is also the case for conformer D2b at an applied pressure of 100 GPa, which is a behavior that has not been observed in the two other investigated studies. We attribute this to the previously described circumstance that the neutral conformer D2a has more flexibility in how it can respond to pressure. We conclude that conformer D2a becomes the most significant conformer at high pressure according to our methodology.

This example shows that the structural details of the investigated conformers are important for the understanding of energetic effects due to pressure. The solvent environment contributing to the stabilization and in some cases to the accessibility of zwitterionic structures is only one aspect that has to be addressed in the discussion. At high pressure, the structural compactness and rigidness of the investigated conformers become essential to the overall energetics of the system. In the case of 2-dimethylaminobenzoic acid, this leads to the described destabilization of the zwitterionic state under pressure, which is not visible in the other examples considered in this paper.

### 4.3 Dimerization of Orthosilicic Acid

In an experimental study conducted by Hunt et al.,^61^ the influence of different parameters, such as temperature or SiO_2_ concentration, on the polymerization and depolymerization of aqueous silica was investigated. With the use of Raman spectroscopy, it could be shown that elevated pressure results in the depolymerization of silica. We chose the dimerization of orthosilicic acid as a modeling system to evaluate the influence of including C-PCM in pressure calculations via GOSTSHYP in the case of reactions. By comparison of the theoretical results depending on the usage of C-PCM with the experimental data, the applicability of the combined GOSTSHYP/C-PCM approach was examined.

The dimerization was simulated at ambient pressure and at 1, 5, and 10 GPa applied with GOSTSHYP. The effects of the implicit chemical environment were analyzed by using C-PCM with the dielectric constant of water and then compared to calculations without the use of an implicit solvation method. In previous theoretical studies dedicated to the dimerization reaction, neutral and anionic mechanisms were proposed.^66,69^ Here, we focus exclusively on the neutral concerted mechanism, shown in Figure 10.

**Figure 10:**
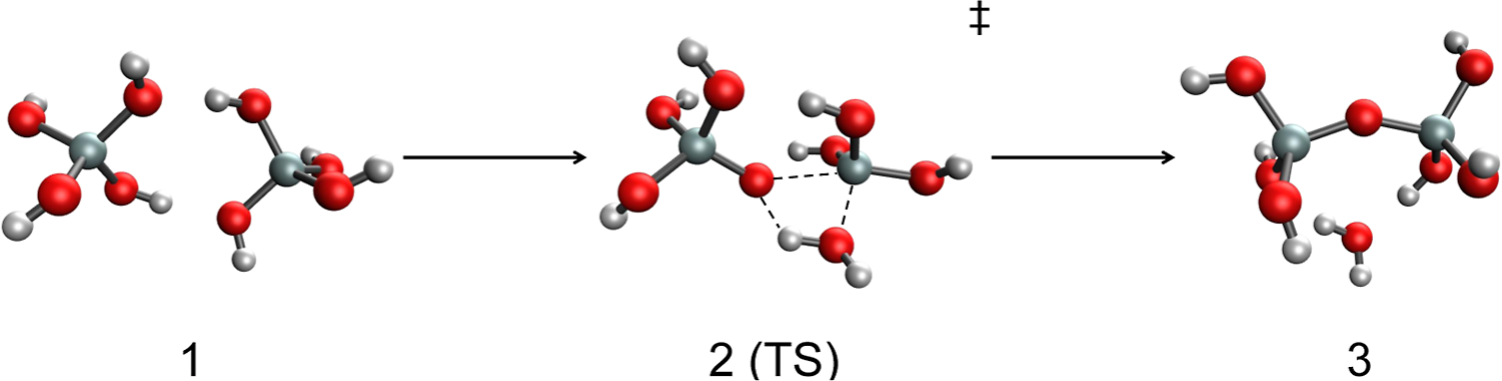
Concerted mechanism for the dimerization of orthosilicic acid calculated on a DFT level of theory (B3LYP-D3­(BJ)/6–311++G­(d,p)/C-PCM) at ambient pressure conditions. (1) Orthosilicic acid educts, (2) transition state (TS), and (3) dimer product.

For the geometries of the educts and the transition state (TS), structures similar to the ones used by Trinh et al.^66^ were constructed. The free energies of the transition state and the products in relation to the free energy of the educts, Δ*G* ^‡^ and Δ*G*, are summarized in Table 3.

**Table 3:** Free Activation energy Δ*G* ^‡^ and the Free Reaction energy Δ*G* of the Dimerization of Orthosilicic Acid Calculated on a DFT (B3LYP-D3­(BJ)/6-311++G­(d,p)) Level of Theory^a^

	Δ*G* ^‡^ / (kcal/mol)		Δ*G*/ (kcal/mol)	
	C-PCM	No C-PCM	C-PCM	No C-PCM
Ambient pressure	29.5	30.1	–5.06	–5.00
1 GPa	30.4	29.4	–4.24	–5.74
5 GPa	26.1	26.5	17.3	–10.6
10 GPa	20.4	20.2	13.0	–9.24

a Pressure at different levels was applied via GOSTSHYP. For each pressure value, the calculations were performed either with C-PCM and a dielectric constant of water or without any solvation model.

Comparing the results for the transition state with regards to the usage of C-PCM, it can be stated that the differences between the obtained values with and without C-PCM for each pressure value are not higher than 1 kcal/mol. In the absence of C-PCM, Δ*G* ^‡^ decreases with increasing pressure. To form the transition state, spatial proximity is required, which is favored at elevated pressure. If C-PCM is used additionally, at a pressure of 1 GPa, Δ*G* ^‡^ increases slightly, indicating that, at this point, the relatively low pressure of 1 GPa has a minor impact compared to the electrostatic interaction simulated with C-PCM. At 1 GPa, the educt of the reaction is notably stabilized by C-PCM compared to calculations without pressure.

The second aspect under consideration is the overall energetics of the dimerization. At ambient pressure, the difference of the Δ*G* values using C-PCM and in the absence of a solvation model is almost negligible. At elevated pressure values of 1 GPa, a larger difference of the reaction free energy can be observed. Without any solvation model, the absolute of Δ*G* becomes larger in magnitude, indicating that the reaction is expected to be more favorable. However, with C-PCM used, a decrease of Δ*G* is visible. Similar to the energetics of the activation energy Δ*G* ^‡^, we attribute this behavior to the relatively high stabilization of the educt when C-PCM is used. Yet, with a difference of 1.5 kcal/mol, the difference of the values obtained at 1 GPa can still be considered as small.

In contrast to the results of the transition state energetics, the overall free reaction energy indicates a substantial difference at higher pressure depending on the usage of C-PCM. At 5 GPa, by just using GOSTSHYP, the free energy of the reaction increases in magnitude and becomes more negative, which means that the reaction is thermodynamically more preferred under high pressure. On the other hand, if C-PCM is used, the calculated Δ*G* value shows an entirely different result. In this case, the Δ*G* value suggests that the product is no longer favored under these circumstances. This is also the case at a higher pressure of 10 GPa. Again, this is a result that can only be achieved by combining GOSTSHYP with C-PCM. Otherwise, even at 10 GPa, the reaction is more favored energetically than at ambient conditions. These divergent results can be explained with the geometries of the product. At high pressure combined with C-PCM, the released water molecule from the reaction cannot leave the reaction site and forms an adduct with the silica dimer, which is shown in Figure 11.

**Figure 11:**
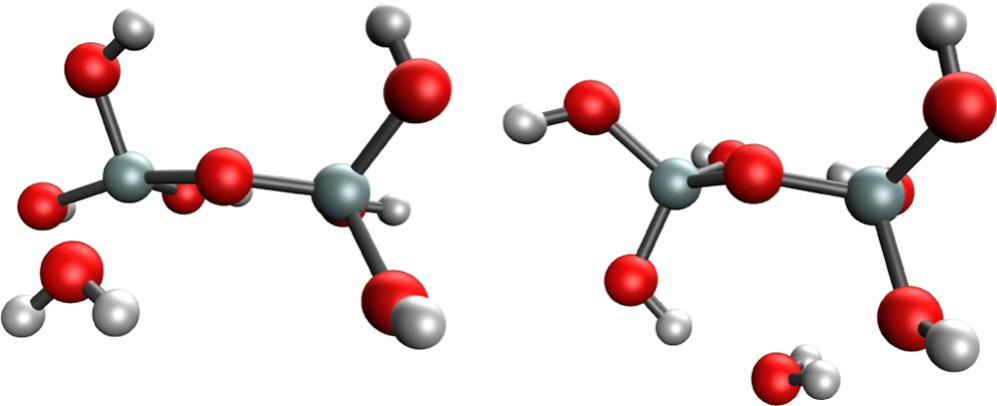
Optimized structures of the products of the dimerization reaction of orthosilicic acid at an applied pressure via GOSTSHYP of 5 GPa combined with C-PCM (left) and without solvation environment (right). A DFT (B3LYP-D3­(BJ)/6-311++G­(d,p)) level of theory was used in both attempts.

The occurrence of hypervalent silicon intermediates is well-known in the literature^122,123^ and could contribute significantly to the molecular geometries under high pressure. Our results for elevated pressure with consideration of an implicit aqueous environment are in qualitative agreement with experimental data from Hunt et al.,^61^ who concluded that increasing pressure leads to the depolymerization of silica at room temperature. The authors supported their conclusion by observing an increase of the Raman peak associated with orthosilicic acid at elevated pressure. The aforementioned behavior can be observed in our calculations based on the change in the sign of the Δ*G* values. This indicates that the combination of GOSTSHYP and C-PCM leads to a significant change of the system’s potential energy surface, resulting in a more physical description of the system under compression. However, it has to be noted that experimental data are limited to 2 GPa. Despite these deviations in the pressure values, the overall trend remains consistent.

## 5 Conclusion and Outlook

In this work, we presented an independent handling of molecular surfaces inside the GOSTSHYP and X-HCFF approaches for quantum chemical pressure simulations. With this standalone routine, a stable way for combining these pressure models with the C-PCM, which includes solvent effects implicitly, could be established inside the Q-Chem quantum chemistry package. This allows the consideration of the chemical environment in these single-molecule pressure models, even with low computational cost. Additionally, the new interface for selecting different ways to represent the molecular surface simplifies the implementation of new surface construction algorithms in the future.

With the new independent surface construction routine, the effects of pressure via GOSTSHYP and X-HCFF on molecular structures, which are unavailable in gas-phase calculations, can now be explored. This was shown to be especially relevant for compounds that can occur in both zwitterionic and neutral structures. For instance, the zwitterionic structure of glycine, which is the most stable one in aqueous solution and in the solid state, can now be investigated under pressure applied by GOSTSHYP combined with C-PCM, improving the physical accuracy of the pressure models. This enables a consistent investigation of how compression alters the balance between intramolecular electrostatics and the environmental stabilization. Comparing the energy values of the neutral and zwitterionic structures of glycine, it could be shown that the energetic preference of the zwitterionic structure increases under pressure, which can be traced back to the more compact zwitterionic structure. At 300 GPa, the zwitterionic structure is compressed to a ring-like structure corresponding to the transition state of the intramolecular hydrogen transfer at ambient conditions. Moreover, experimentally observed changes of the Raman shifts corresponding to significant vibrational modes could be reproduced in good agreement for zwitterionic glycine using X-HCFF or GOSTSHYP combined with C-PCM. Vibrational frequency analyses under pressure applied by GOSTSHYP were reported for the first time in this work. However, the approach shows its limitations for vibrations that are highly affected by intermolecular interactions.

For sulfamic acid, another compound occurring in both zwitterionic and neutral states, it could be concluded that the use of C-PCM stabilizes the zwitterionic state in a wide range of pressures compared to calculations without a solvent environment regardless of the considered conformer. Moreover, the energetic difference between the zwitterionic and neutral states shows different trends under pressure depending on the usage of C-PCM. In the case of the last considered compound, 2-dimethylaminobenzoic acid, the stabilization of the zwitterion compared to the investigated neutral state conformers decreases under pressure. Hence, it can be concluded that the combination of C-PCM with GOSTSHYP can unveil an interplay of the electrostatic and steric aspects of compounds under pressure.

This is also valid for the investigation of the dimerization of orthosilicic acid. When combining GOSTSHYP with C-PCM, the geometries of the product differ significantly from pure GOSTSHYP calculations, and the free energy values indicate the energetic disfavor of the polymerization reaction. This shows that a combined GOSTSHYP/C-PCM approach can lead to better agreement with experimental findings. Therefore, the analysis of pressure-dependent reaction free energies for this system demonstrates that our approach improves the thermodynamic accuracy of theoretical models under high-pressure conditions. This is particularly relevant for geochemical processes and fundamental physicochemical phenomena. Still, the presented results can only serve as a first step for a more detailed examination of the reaction. The influence of the dielectric constant for this reaction has to be explored, especially considering the pressure dependence of water’s dielectric properties. Although the pressure dependence has only minor effects in the case of glycine, its influence should additionally be tested for the dimerization reaction. Also, other reaction paths have to be included in future studies.

These examples show that the use of C-PCM in GOSTSHYP calculations can provide a more detailed insight into molecules under the influence of an external solvation environment. The combination of single-molecule pressure methods with implicit solvation serves as a first step to include the environment of a molecule in X-HCFF and GOSTSHYP. This made both models suitable for structures that could not be accessed otherwise. By extending the set of accessible model systems, this work enables the investigation of charge-separated biomolecules under extreme deep-Earth or extraterrestrial conditions, highlighting pressure-dependent effects. In the future, explicit intermolecular interactions under pressure need to be considered in detail, with the simultaneous application of C-PCM enabling an implicit representation of outer solvation shells. Especially microsolvation approaches, such as the one proposed by Steiner et al.,^124^ can provide a way to simulate sophisticated explicit chemical environments. Thus, a clear distinction between intramolecular and intermolecular effects on energetic and structural changes at an elevated pressure can be established. Additionally, the investigation of excited states under pressure can benefit from this approach.^125^ With that, the accuracy of GOSTSHYP and X-HCFF compared with experimental results is expected to increase further.

## Supplementary Material



## Notes

Energy (*E*_h_) and RMS gradient (*E*_h_/*a*_0_) values for water, ammonia and benzene including Cartesian coordinates for the purpose of code verification; Raman spectra of zwitterionic glycine under pressure for GOSTSHYP and X-HCFF; and Cartesian coordinates of all investigated conformers of glycine, sulfamic acid, and 2-dimethylaminobenzoic acid, as well as of all critical points for the dimerization of orthosilicic acid (with and without pressure application) (ZIP)
