# Supplementary material for: Enhancing Molecular High-Pressure Simulations by Implicit Solvation
Source: J Phys Chem A. 2026 May 26;130(22):4198–212. doi: 10.1021/acs.jpca.6c02133 (PMC13244558; doi:10.1021/acs.jpca.6c02133)
Supplement: Supplementary file 1 [file jp6c02133_si_001.zip › SI.pdf]

Supporting Information

Enhancing Molecular High-Pressure Simulations by Implicit Solvation

Nico Kißing,[1] Felix Zeller[1] and Tim Neudecker[1,2,3,*]

[1] University of Bremen, Institute for Physical and Theoretical Chemistry, Leobener Straße 6, D-28359 Bremen, Germany

[2] Bremen Center for Computational Materials Science, University of Bremen, Am Fallturm 1, D-28359 Bremen, Germany

[3] MAPEX Center for Materials and Processes, University of Bremen, Bibliothekstraße 1, D-28359 Bremen, Germany

[*]Author to whom correspondence should be addressed: neudecker@uni-bremen.de

1 Code Verification for the Independent Tessellation Routine

The code verification was carried out as described by performing single-point and force calculations on a Hartree-Fock (HF)[1–3] level of theory with the 6-31G basis set.[4] "distort" indicates the usage of the independent tessellation routine in the eXtended Hydrostatic Compression Force Field (X-HCFF)[5] and in Gaussians On Surface Tesserae Simulate HYdrostatic Pressure (GOSTSHYP)[6] available for the development version of Q-Chem 6.2.[7] "scrf" refers to the previously used tessellation method[8–10] which was originally designed for the Polarizable Continuum Model (PCM),[11,12] here specifically the Conductor-like Polarizable Continuum Model (C-PCM).[13,14] A selection of the routine is only available for the pressure models.

Table S1: The obtained energies of water in different test cases. It is noted with “n/a” if a calculation with the “scrf” routine or Q-Chem 6.0 is not possible due to the discussed incompatibilities. A double dashed line separates the sections in which the obtained values should match.

	Energy / E_h		
	“distort”	“scrf”	Q-Chem 6.0
No models used	−75.98157179		−75.98157180
X-HCFF ($p = 0\,\mathrm{MPa}$)	−75.98157179	not tested	not tested
GOSTSHYP ($p = 0\,\mathrm{MPa}$)	−75.98157179	not tested	not tested
C-PCM ($\epsilon = 1$)	-75.98157180		not tested
X-HCFF ($p = 0\,\mathrm{MPa}$), C-PCM ($\epsilon = 1$)	−75.98157180	−75.98157180	−75.98157180
X-HCFF ($p = 50\,\mathrm{GPa}$)	−75.98157179	−75.98157179	−75.98157180
X-HCFF ($p = 50\,\mathrm{GPa}$), C-PCM ($\epsilon = 1$)	−75.98157180	−75.98157180	−75.98157180
GOSTSHYP ($p = 0\,\mathrm{MPa}$), C-PCM ($\epsilon = 1$)	−75.98157180	n/a	n/a
GOSTSHYP ($p = 50\,\mathrm{GPa}$)	−75.87478873	−75.87478873	−75.87478874
GOSTSHYP ($p = 50\,\mathrm{GPa}$), C-PCM ($\epsilon = 1$)	−75.87478874	n/a	n/a
C-PCM ($\epsilon = 1.001$)	−75.98158414		−75.98158414
X-HCFF ($p = 0\,\mathrm{MPa}$), C-PCM ($\epsilon = 1.001$)	−75.98158414	n/a	n/a
X-HCFF ($p = 50\,\mathrm{GPa}$), C-PCM ($\epsilon = 1.001$)	−75.98158414	n/a	n/a
GOSTSHYP ($p = 0\,\mathrm{MPa}$), C-PCM ($\epsilon = 1.001$)	−75.98158414	n/a	n/a
GOSTSHYP ($p = 50\,\mathrm{GPa}$), C-PCM ($\epsilon = 1.001$)	−75.87480249	n/a	n/a

Table S2: The obtained energies of ammonia in different test cases. It is noted with "n/a" if a calculation with the "scrf" routine or Q-CHEM 6.0 crashed due to the discussed incompatibilities. A double dashed line separates the sections in which the obtained values should match.

	Energy / E_h		
	"distort"	"scrf"	Q-CHEM 6.0
No models used	−56.16132547		−56.16132547
X-HCFF ($p = 0\,\mathrm{MPa}$)	−56.16132547	not tested	not tested
GOSTSHYP ($p = 0\,\mathrm{MPa}$)	−56.16132547	not tested	not tested
C-PCM ($\epsilon = 1$)	−56.16132547		not tested
X-HCFF ($p = 0\,\mathrm{MPa}$),<br>C-PCM ($\epsilon = 1$)	−56.16132547	−56.16132547	−56.16132547
X-HCFF ($p = 50\,\mathrm{GPa}$)	−56.16132547	−56.16132547	−56.16132547
X-HCFF ($p = 50\,\mathrm{GPa}$),<br>C-PCM ($\epsilon = 1$)	−56.16132547	−56.16132547	−56.16132547
GOSTSHYP ($p = 0\,\mathrm{MPa}$),<br>C-PCM ($\epsilon = 1$)	−56.16132547	n/a	n/a
GOSTSHYP ($p = 50\,\mathrm{GPa}$)	−56.03129241	−56.03129241	−56.03129241
GOSTSHYP ($p = 50\,\mathrm{GPa}$),<br>C-PCM ($\epsilon = 1$)	−56.03129241	n/a	n/a
C-PCM ($\epsilon = 1.001$)	−56.16133468		−56.16133468
X-HCFF ($p = 0\,\mathrm{MPa}$),<br>C-PCM ($\epsilon = 1.001$)	−56.16133468	n/a	n/a
X-HCFF ($p = 50\,\mathrm{GPa}$),<br>C-PCM ($\epsilon = 1.001$)	−56.16133468	n/a	n/a
GOSTSHYP ($p = 0\,\mathrm{MPa}$),<br>C-PCM ($\epsilon = 1.001$)	−56.16133468	n/a	n/a
GOSTSHYP ($p = 50\,\mathrm{GPa}$),<br>C-PCM ($\epsilon = 1.001$)	−56.03130255	n/a	n/a

Table S3: The obtained energies of benzene in different test cases. It is noted with "n/a" if a calculation with the "scrf" routine or Q-Chem 6.0 crashed due to the discussed incompatibilities. A double dashed line separates the sections in which the obtained values should match.

	Energy / E_h		
	"distort"	"scrf"	Q-Chem 6.0
No models used	−230.6225183		−230.6225183
X-HCFF ($p = 0\,\mathrm{MPa}$)	−230.6225183	not tested	not tested
GOSTSHYP ($p = 0\,\mathrm{MPa}$)	−230.6225183	not tested	not tested
C-PCM ($\epsilon = 1$)	−230.6225183		not tested
X-HCFF ($p = 0\,\mathrm{MPa}$), C-PCM ($\epsilon = 1$)	−230.6225183	−230.6225183	−230.6225183
X-HCFF ($p = 50\,\mathrm{GPa}$)	−230.6225183	−230.6225183	−230.6225183
X-HCFF ($p = 50\,\mathrm{GPa}$), C-PCM ($\epsilon = 1$)	−230.6225183	−230.6225183	−230.6225183
GOSTSHYP ($p = 0\,\mathrm{MPa}$), C-PCM ($\epsilon = 1$)	−230.6225183	n/a	n/a
GOSTSHYP ($p = 50\,\mathrm{GPa}$)	−230.2584894	−230.2584894	−230.2584894
GOSTSHYP ($p = 50\,\mathrm{GPa}$), C-PCM ($\epsilon = 1$)	−230.2584894	n/a	n/a
C-PCM ($\epsilon = 1.001$)	−230.6225225		−230.6225225
X-HCFF ($p = 0\,\mathrm{MPa}$), C-PCM ($\epsilon = 1.001$)	−230.6225225	n/a	n/a
X-HCFF ($p = 50\,\mathrm{GPa}$), C-PCM ($\epsilon = 1.001$)	−230.6225225	n/a	n/a
GOSTSHYP ($p = 0\,\mathrm{MPa}$), C-PCM ($\epsilon = 1.001$)	−230.6225225	n/a	n/a
GOSTSHYP ($p = 50\,\mathrm{GPa}$), C-PCM ($\epsilon = 1.001$)	−230.2584952	n/a	n/a

Table S4: The obtained Root Mean Square (RMS) gradients of water in different test cases. It is noted with "n/a" if a calculation with the "scrf" routine or Q-CHEM 6.0 is not possible due to the discussed incompatibilities. A double dashed line separates the sections in which the obtained values should match.

<table>
<tr><th></th><th colspan="3">RMS gradient / (E_h/a_0)</th></tr>
<tr><th></th><th>"distort"</th><th>"scrf"</th><th>Q-CHEM 6.0</th></tr>
<tr><td>No models used</td><td colspan="2">0.02513</td><td>0.02513</td></tr>
<tr><td>X-HCFF ($p = 0\,\mathrm{MPa}$)</td><td>0.02513</td><td>not tested</td><td>not tested</td></tr>
<tr><td>GOSTSHYP ($p = 0\,\mathrm{MPa}$)</td><td>0.02513</td><td>not tested</td><td>not tested</td></tr>
<tr><td>C-PCM ($\epsilon = 1$)</td><td colspan="2">0.02513</td><td>not tested</td></tr>
<tr><td>X-HCFF ($p = 0\,\mathrm{MPa}$),
C-PCM ($\epsilon = 1$)</td><td>0.02513</td><td>0.02513</td><td>0.02513</td></tr>
<tr><td>X-HCFF ($p = 50\,\mathrm{GPa}$)</td><td>0.04403</td><td>0.04403</td><td>0.04403</td></tr>
<tr><td>X-HCFF ($p = 50\,\mathrm{GPa}$),
C-PCM ($\epsilon = 1$)</td><td>0.04403</td><td>0.04403</td><td>0.04403</td></tr>
<tr><td>GOSTSHYP ($p = 0\,\mathrm{MPa}$),
C-PCM ($\epsilon = 1$)</td><td>0.02513</td><td>n/a</td><td>n/a</td></tr>
<tr><td>GOSTSHYP ($p = 50\,\mathrm{GPa}$)</td><td>0.03624</td><td>0.03624</td><td>0.03624</td></tr>
<tr><td>GOSTSHYP ($p = 50\,\mathrm{GPa}$),
C-PCM ($\epsilon = 1$)</td><td>0.03624</td><td>n/a</td><td>n/a</td></tr>
<tr><td>C-PCM ($\epsilon = 1.001$)</td><td colspan="2">0.02513</td><td>0.02513</td></tr>
<tr><td>X-HCFF ($p = 0\,\mathrm{MPa}$),
C-PCM ($\epsilon = 1.001$)</td><td>0.02513</td><td>n/a</td><td>n/a</td></tr>
<tr><td>X-HCFF ($p = 50\,\mathrm{GPa}$),
C-PCM ($\epsilon = 1.001$)</td><td>0.04404</td><td>n/a</td><td>n/a</td></tr>
<tr><td>GOSTSHYP ($p = 0\,\mathrm{MPa}$),
C-PCM ($\epsilon = 1.001$)</td><td>0.02513</td><td>n/a</td><td>n/a</td></tr>
<tr><td>GOSTSHYP ($p = 50\,\mathrm{GPa}$),
C-PCM ($\epsilon = 1.001$)</td><td>0.03625</td><td>n/a</td><td>n/a</td></tr>
</table>

Table S5: The obtained RMS gradients of ammonia in different test cases. It is noted with "n/a" if a calculation with the "scrf" routine or Q-CHEM 6.0 is not possible due to the discussed incompatibilities. A double dashed line separates the sections in which the obtained values should match.

	RMS gradient / (E_h/a_0)		
	"distort"	"scrf"	Q-CHEM 6.0
No models used	0.01721		0.01721
X-HCFF ($p = 0\,\mathrm{MPa}$)	0.01721	not tested	not tested
GOSTSHYP ($p = 0\,\mathrm{MPa}$)	0.01721	not tested	not tested
C-PCM ($\epsilon = 1$)	0.01721		not tested
X-HCFF ($p = 0\,\mathrm{MPa}$), C-PCM ($\epsilon = 1$)	0.01721	0.01721	0.01721
X-HCFF ($p = 50\,\mathrm{GPa}$)	0.03455	0.03455	0.03455
X-HCFF ($p = 50\,\mathrm{GPa}$), C-PCM ($\epsilon = 1$)	0.03455	0.03455	0.03455
GOSTSHYP ($p = 0\,\mathrm{MPa}$), C-PCM ($\epsilon = 1$)	0.01721	n/a	n/a
GOSTSHYP ($p = 50\,\mathrm{GPa}$)	0.02638	0.02638	0.02638
GOSTSHYP ($p = 50\,\mathrm{GPa}$), C-PCM ($\epsilon = 1$)	0.02638	n/a	n/a
C-PCM ($\epsilon = 1.001$)	0.01721		0.01721
X-HCFF ($p = 0\,\mathrm{MPa}$), C-PCM ($\epsilon = 1.001$)	0.01721	n/a	n/a
X-HCFF ($p = 50\,\mathrm{GPa}$), C-PCM ($\epsilon = 1.001$)	0.03456	n/a	n/a
GOSTSHYP ($p = 0\,\mathrm{MPa}$), C-PCM ($\epsilon = 1.001$)	0.01721	n/a	n/a
GOSTSHYP ($p = 50\,\mathrm{GPa}$), C-PCM ($\epsilon = 1.001$)	0.02638	n/a	n/a

Table S6: The obtained RMS gradients of benzene in different test cases. It is noted with "n/a" if a calculation with the "scrf" routine or Q-CHEM 6.0 is not possible due to the discussed incompatibilities. A double dashed line separates the sections in which the obtained values should match.

	RMS gradient / (E_h/a_0)		
	"distort"	"scrf"	Q-CHEM 6.0
No models used	0.007940		0.007940
X-HCFF ($p = 0\,\mathrm{MPa}$)	0.007940	not tested	not tested
GOSTSHYP ($p = 0\,\mathrm{MPa}$)	0.007940	not tested	not tested
C-PCM ($\epsilon = 1$)	0.007940		not tested
X-HCFF ($p = 0\,\mathrm{MPa}$), C-PCM ($\epsilon = 1$)	0.007940	0.007940	0.007940
X-HCFF ($p = 50\,\mathrm{GPa}$)	0.01914	0.01914	0.01914
X-HCFF ($p = 50\,\mathrm{GPa}$), C-PCM ($\epsilon = 1$)	0.01914	0.01914	0.01914
GOSTSHYP ($p = 0\,\mathrm{MPa}$), C-PCM ($\epsilon = 1$)	0.007940	n/a	n/a
GOSTSHYP ($p = 50\,\mathrm{GPa}$)	0.01315	0.01315	0.01315
GOSTSHYP ($p = 50\,\mathrm{GPa}$), C-PCM ($\epsilon = 1$)	0.01315	n/a	n/a
C-PCM ($\epsilon = 1.001$)	0.007942		0.007942
X-HCFF ($p = 0\,\mathrm{MPa}$), C-PCM ($\epsilon = 1.001$)	0.007942	n/a	n/a
X-HCFF ($p = 50\,\mathrm{GPa}$), C-PCM ($\epsilon = 1.001$)	0.01914	n/a	n/a
GOSTSHYP ($p = 0\,\mathrm{MPa}$), C-PCM ($\epsilon = 1.001$)	0.007942	n/a	n/a
GOSTSHYP ($p = 50\,\mathrm{GPa}$), C-PCM ($\epsilon = 1.001$)	0.01315	n/a	n/a

2 Raman Spectra of Zwitterionic Glycine under Pressure

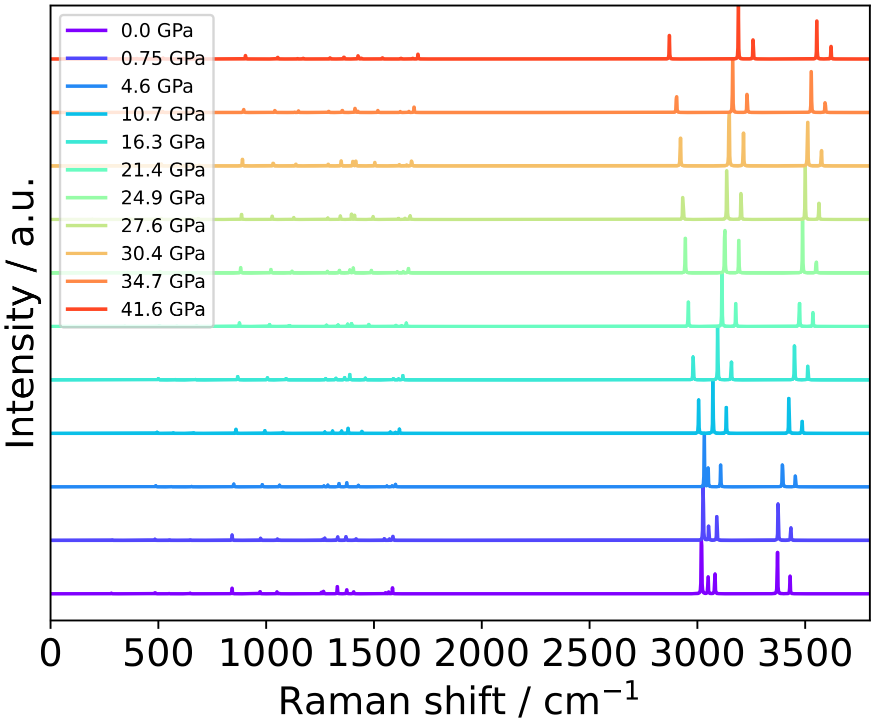

Figure S1: The Raman spectrum of zwitterionic glycine for different pressures applied by GOSTSHYP using Density Functional Theory (DFT)[15,16] with a B3LYP-D3(BJ)/aug-cc-pVDZ/C-PCM level of theory.

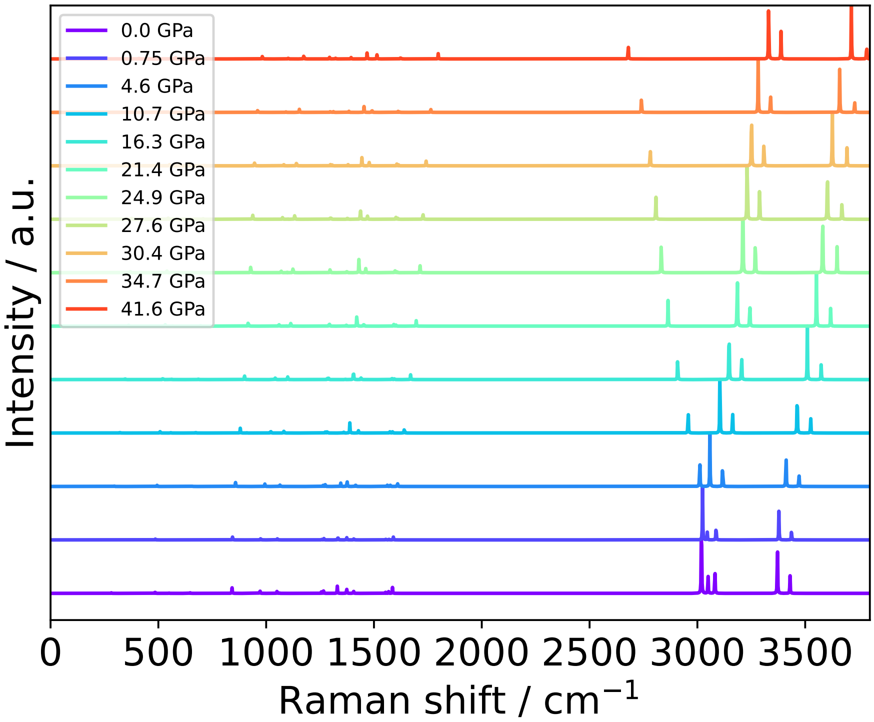

Figure S2: The Raman spectrum of zwitterionic glycine for different pressures applied by X-HCFF using DFT (B3LYP-D3(BJ)/aug-cc-pVDZ/C-PCM).

3 References

(1) Hartree, D. R. *Math. Proc. Cambridge Philos. Soc.* **1928**, *24*, 111–132.

(2) Fock, V. *Z. Phys.* **1930**, *61*, 126–148.

(3) Hartree, D. R.; Hartree, W. *Proc. R. Soc. London A - Math Phys. Sci.* **1935**, *150*, 9–33.

(4) Hehre, W. J.; Ditchfield, R.; Pople, J. A. *J. Chem. Phys.* **1972**, *56*, 2257–2261.

(5) Stauch, T. *J. Chem. Phys.* **2020**, *153*, 134503.

(6) Scheurer, M.; Dreuw, A.; Epifanovsky, E.; Head-Gordon, M.; Stauch, T. *J. Chem. Theory Comput.* **2021**, *17*, 583–597.

(7) Epifanovsky, E.; Gilbert, A. T. B.; Feng, X.; Lee, J.; Mao, Y.; Mardirossian, N.; Pokhilko, P.; White, A. F.; Coons, M. P.; Dempwolff, A. L., et al. *J. Chem. Phys.* **2021**, *155*, 084801.

(8) Lange, A. W.; Herbert, J. M. *J. Chem. Phys.* **2010**, *133*, 244111.

(9) Lange, A. W.; Herbert, J. M. *Chem. Phys. Lett.* **2011**, *509*, 77–87.

(10) Herbert, J. M. *WIREs Comput. Mol. Sci.* **2021**, *11*, e1519.

(11) Miertuš, S.; Scrocco, E.; Tomasi, J. *Chem. Phys.* **1981**, *55*, 117–129.

(12) Tomasi, J.; Mennucci, B.; Cammi, R. *Chem. Rev.* **2005**, *105*, 2999–3094.

(13) Barone, V.; Cossi, M. *J. Phys. Chem. A* **1998**, *102*, 1995–2001.

(14) Cossi, M.; Rega, N.; Scalmani, G.; Barone, V. *J. Comput. Chem.* **2003**, *24*, 669–681.

(15) Hohenberg, P.; Kohn, W. *Phys. Rev.* **1964**, *136*, B864–B871.

(16) Kohn, W.; Sham, L. J. *Phys. Rev.* **1965**, *140*, A1133–A1138.
